# Rapid Gene Expression Changes in Peripheral Blood Lymphocytes upon Practice of a Comprehensive Yoga Program

**DOI:** 10.1371/journal.pone.0061910

**Published:** 2013-04-17

**Authors:** Su Qu, Solveig Mjelstad Olafsrud, Leonardo A. Meza-Zepeda, Fahri Saatcioglu

**Affiliations:** 1 Department of Biosciences, University of Oslo, Oslo, Norway; 2 Department of Tumor Biology, Institute for Cancer Research, Oslo University Hospital, Oslo, Norway; 3 Genomics Core Facility, Department of Biosciences, University of Oslo, Oslo, Norway; South Texas Veterans Health Care System and University of Texas Health Science Center at San Antonio, United States of America

## Abstract

One of the most common integrative medicine (IM) modalities is yoga and related practices. Previous work has shown that yoga may improve wellness in healthy people and have benefits for patients. However, the mechanisms of how yoga may positively affect the mind-body system are largely unknown. Here we have assessed possible rapid changes in global gene expression profiles in the peripheral blood mononuclear cells (PBMCs) in healthy people that practiced either a comprehensive yoga program or a control regimen. The experimental sessions included gentle yoga postures, breathing exercises, and meditation (Sudarshan Kriya and Related Practices – SK&P) compared with a control regimen of a nature walk and listening to relaxing music. We show that the SK&P program has a rapid and significantly greater effect on gene expression in PBMCs compared with the control regimen. These data suggest that yoga and related practices result in rapid gene expression alterations which may be the basis for their longer term cell biological and higher level health effects.

## Introduction

Integrative medicine (IM) approaches have gained significant interest in recent years in search of alternative solutions to the health care challenges we face today [1] [2]. IM in particular focuses on preventive maintenance of health with emphasis on diet, lifestyle, stress management, and emotional well-being [3]. In addition to the latest scientific findings and evidence based approaches, IM taps on time-tested traditional modalities to increase health and wellness, as well as helping treat disease states.

One of the most common IM approaches is yoga and associated practices, estimated to be a 5000-year-old discipline originating from India [4]. Yoga is divided into several branches, but the one that is most popular in the West is Hatha-yoga, which includes control of posture (asanas) and the manipulation of respiration (pranayama). Hatha-yoga is considered to improve bodily functions through the manipulation of cardiovascular, respiratory, metabolic, and other control mechanisms (e.g. [5] [6] [7]). Whereas asanas are generally familiar, pranayamas and the central role that they have in yoga are not well appreciated in the West.

One of the most widely used breathing programs derived from yoga is Sudarshan Kriya (SK) (for descriptions, see [8], [9]. SK is traditionally understood to use specific rhythms of the breath to eliminate stress, support the various organs and systems within the body, transform overpowering emotions and restore peace of mind. Recent research on SK and related practices (SK&P), which include yoga asanas, pranayama, SK and meditative components, has indicated significant effects on various aspects of the physiology and the psychology of the participants. For example, SK&P was found to have antidepressant effects in clinical settings and was comparable to the antidepressant drug Imipramine in its efficacy [8]. Another study found significantly lower levels of blood lactate in practitioners of SK&P compared with the control group [10]. Conversely, the levels of superoxide dismutase (SOD), catalase, and glutathione, three major defenses against oxidative stress, were all found to be significantly higher in SK&P practitioners compared with the control group [10]. These data suggested that people who practice SK&P have an improved antioxidant status and defense against oxidative stress.

Effect of SK&P on psychological health is not limited to clinical patients. For example, a recent controlled study found that participants in the SK&P group, but not the control group, significantly lowered their degree of anxiety, depression and stress, and also increased their degree of optimism [9]. Various additional studies indicate that SK&P results in improved physical and psychological health and well being. For example, SK&P appears to affect brain function, with EEG changes indicative of a state of wakeful alertness [11].

Despite these and similar intriguing findings for other branches of yoga, the molecular and cell biological mechanisms of the effects of yoga and related practices remain largely unknown. It is now well established that perturbations in the environment give rise to distinct changes in gene expression. Recent work has shown that not only physical changes in the environment, but also psychological, social, and cultural components can induce gene expression changes, studied by the emerging field of psychosocial genomics (for a review, see [12]). For example, adverse life experiences have been suggested to give rise to significant changes in gene expression in circulating immune cells (for a review, see [13]). Consistent with this framework, the first set of studies on long-term (months to years) yogic/meditative practitioners have found that these practices may positively affect gene expression profiles in immune cells in the circulation [14] [15] [16] [17]. We hypothesized that yogic practices may actually give rise to rapid gene expression changes in PBMCs immediately upon practice. Specific aim of the study was to assess whether such changes occur. Here we present data which show that there are rapid and significant changes in gene expression in PBMCs upon practice of SK&P compared with a control regimen.

## Materials and Methods

### Study Design and Subjects

The study determined immediate effects of a comprehensive yoga program compared with a control regimen on gene expression profiles in PBMCs. During 4 consecutive days, at the same time of the day (6.30 am – 8.30 am), subjects either practiced SK&P or participated in a control regimen composed of a nature walk (to emulate the yoga asanas part of SK&P) and listening to relaxing music (to emulate the relaxation/meditation part of the session). Each subject participated in two experimental and two control regimens, one intervention per day, on four consecutive days, at the same time of the day (days 1 and 2 SK&P, days 3 and 4 control regimen; see Figure S1 for an overview). Right before and after each regimen, 20 ml blood was drawn, PBMCs were immediately isolated, total RNA was recovered and used in gene expression profiling experiments interrogating >47,000 independent transcripts in two hybridization runs.

The study originally included 14 participants who were attending a one-week yoga retreat in Oppenau, Germany. The samples from 4 participants could not be used since either they missed a session, they could not donate enough blood, or the RNA quality was not good enough for further analysis. Participants had previously, before coming to the retreat, learned SK&P, a comprehensive yoga and yogic breathing program (see [9], for details of the program) and have been practicing it regularly for 1,5 months to 5 years. The participants were recruited to the study through an announcement during the first day of the retreat and all signed a consent form. Inclusion criteria were: male, age 18–50 years, no chronic disease, and good psychological health (confirmed by General Health Questionnaire 28 (GHQ28), mean score 13, data not shown).

### Ethics Statement

The study was approved by the South East Health Region Ethics Committee in Oslo prior to the commencement of experiments (REK S-09246). Written informed consent was obtained from all subjects before the first day of experimentation.

### Intervention

Participants took part in the following routine 4 days in a row from 6:30 am to 8:30 am: The first two days they practiced SK&P which includes gentle stretches (yoga postures), specific breathing exercises (pranayamas and SK), ending with a meditative experience (for detailed descriptions, see [9]). The sessions were led by experienced instructors in SK&P in a standard format. For days 3 and 4, at the same time period, participants went for a nature walk for 60 min and then listened to classical or relaxing jazz music in silence for 60 min in the same room where they had the SK&P practice. Immediately before and right after the interventions, 20 ml of blood was collected by venous puncture from each participant (8 times in total) and processed as described below.

### RNA isolation

Blood was collected in tubes containing EDTA as anticoagulent (Greiner Bio-One), immediately loaded onto LeukoLOCK RNA isolation system (Ambion) and processed according to the manufacturer’s recommendations. Peripheral blood lymphocytes (PBMCs) captured on filters were immediately frozen at –20°C for parallel processing of subsequent steps. Cells were then lysed on the filters, RNA was eluted and precipitated using the TRI Reagent (Ambion) according to the manufacturer’s recommendations. RNA concentrations were determined and a sample was run on an agarose gel to determine RNA quality.

### Microarray analysis

For each individual sample, 500 ng of total RNA was used with the Illumina TotalPrep Amplification Kit (Ambion) to make biotin-labelled, amplified cRNA which were then hybridized in two batches to HumanWG-6 v3 Expression BeadChips (Illumina) enabling profiling of >47,000 transcripts. The Illumina arrays were scanned with the BeadArray reader, and data extraction and initial quality control was performed in GenomeStudio version 2010.1 using Gene Expression module v.1.6.0 (Illumina). Additional quality control as well as statistical analysis was performed using J-Express 2009 [18]. An agglomerative hierarchical clustering as implemented in J-Express were performed on the full dataset to investigate global trends in the data. A Principal Component Analysis (PCA) [19] plot is presented in Figure S2 which indicated that there was a tendency towards samples from the same subject to cluster together, as would be expected.

Both SAM (Significance Analysis of Microarrays) [20] and Rank Product analysis [21] were utilized for further analysis where each of the four interventions were considered as individual experiments, and thus each type of intervention was repeated twice. Paired SAM analysis was performed by comparing yoga day 1 before treatment vs yoga day 1 after treatment for each subject and similarly repeated on all the interventions. This produced four sorted lists of differentially expressed genes with a threshold q-value <20%, two for each identical intervention (replicates). The q-value represents the adjusted p-value found using an optimized FDR approach [20]. To investigate consistencies between the two lists for each of the interventions, Rank Product analysis was performed comparing the ranks of the top differentially expressed genes for SK&P day 1 vs the ranks of the top differentially expressed genes for SK&P day 2, and similarly the ranks of the top differentially expressed genes for control day 3 vs the ranks of the top differentially expressed genes for control day 4, respectively. The threshold was set at q-value <10%. The fold-change for the resulting gene lists among the SK&P and control conditions was calculated by obtaining the ratio of normalized raw expression values for each replicate subject treatment before and after the SK&P or the control regimen. Subsequently, the ratio of ratios was calculated by determining the ratio between treatments within each individual, SK&P vs control. Finally the average of the ratios of ratios was calculated among all individuals for each gene. GEO accession number for the microarray data is GSE44777.

### Quantitative PCR analysis

From the gene lists generated in the above analysis, some in the top 20 that are differentially regulated either in the SK&P or the control condition were chosen and quantitative PCR (qPCR) analysis was performed as described before [22] on 8 individual samples per intervention (evenly distributed among the available samples). Primer sequences are presented in Figure S3.

### Statistics

For qPCR analysis, comparisons were made with the Student's t test. A value of *P*<0.05 indicated statistical significance.

## Results

Based on previous findings of yogic practices on various psychological and physiological parameters (for reviews, see [5], [23]), and the recent studies indicating long-term molecular effects [14] [15] [16] [17], we hypothesized that these practices may have measurable rapid effects on gene expression patterns in circulating immune cells of participants. The current study was conducted to test the hypothesis that SK&P can have rapid effects on gene expression in PBMCs. To that end, SK&P practitioners who were on a week-long yoga retreat were recruited to the study. During 4 consecutive days, subjects either practiced SK&P or participated in a control regimen composed of a nature walk and listening to relaxing music. Right before and after each regimen, 20 ml blood was drawn, PBMCs were immediately isolated, total RNA was recovered and used in gene expression profiling experiments.

To calculate differential gene expression that may be induced by the two regimens, SK&P compared with control, we first used SAM (Significance Analysis of Microarrays) [20] for pairwise comparisons (before and after each regimen for each subject and intervention). To investigate the consistency between the two replicas of the identical treatments, a meta-analysis of the ranked lists was performed using Rank Product [21]. This analysis determined whether the same gene/probe sets had similar rank in the lists generated from the two identical interventions per subject. The result from this analysis is presented in Table S1.

**Figure 1:**
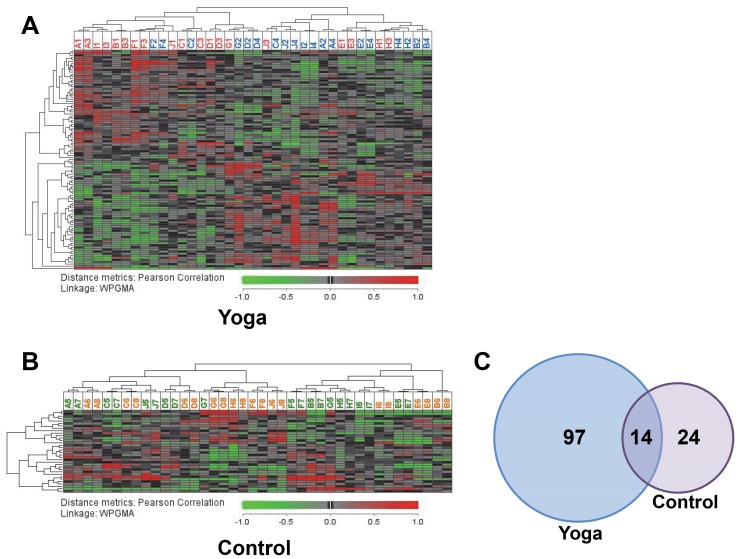
Heatmaps of the differentially expressed genes induced by the yoga (A) and control (B) regimens. Genes were clustered as described in Materials and Methods. Letters in columns represent subjects and the numbers after the letters refer to the interventions (1 and 3  =  before yoga; 2 and 4  =  after yoga; 5 and 7 before the control regimen; 6 and 8, after the control regimen) (also see Figure S1). C) Venn diagram indicating the overlap in the genes commonly regulated by the yoga and control regimens.

**Figure 2:**
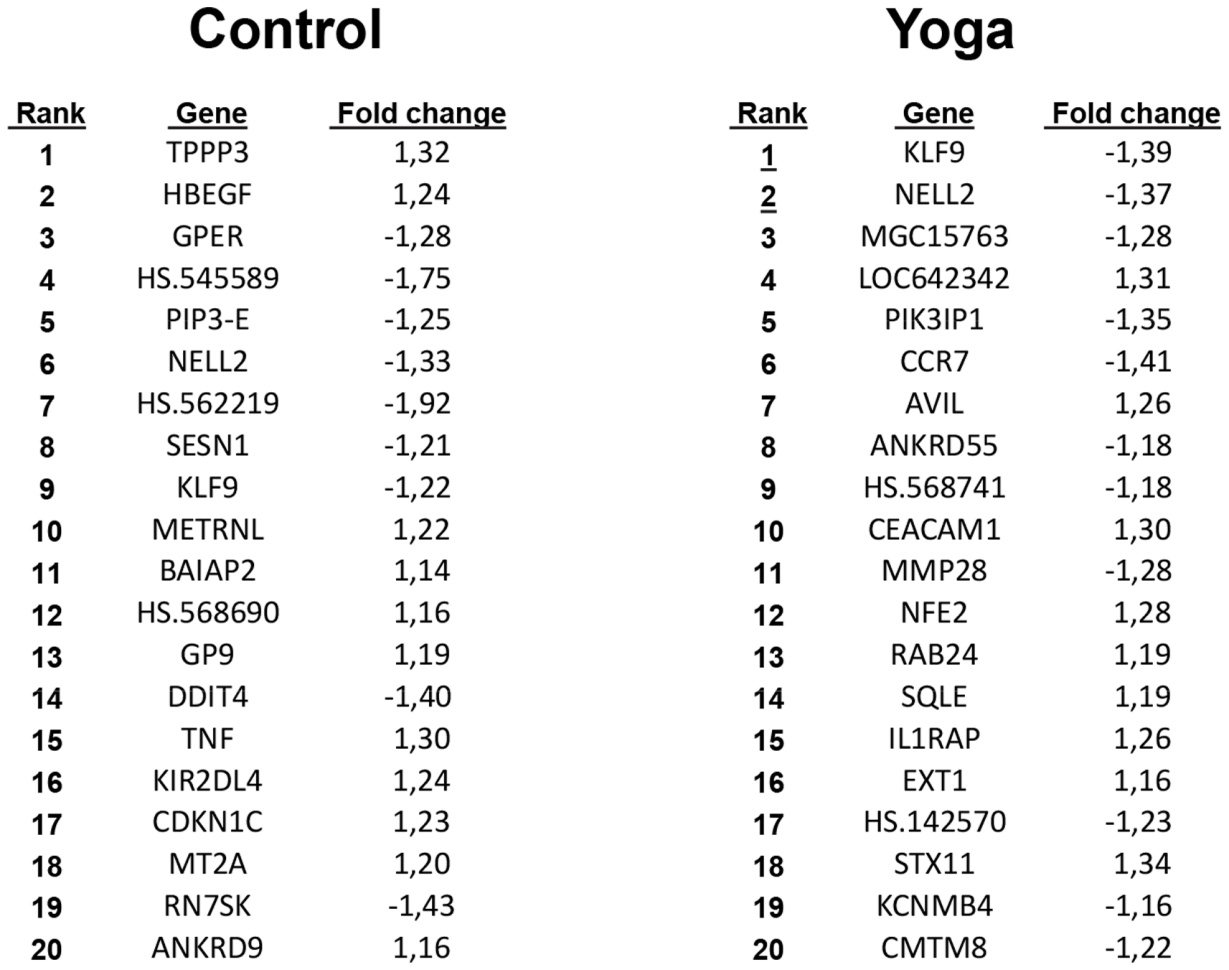
The top 20 ranked genes that are differentially expressed upon yoga (SK&P) or the control regimen. The ranks and the fold changes were determined as described in Materials and Methods. Values greater than 1.0 denote an increase and values smaller than –1.0 indicate a decrease in gene expression.

Using the identified differentially expressed genes, we performed hierarchical clustering using distance metrics (Pearson correlation and complete linkage, Weighted Pair Group Method with Arithmetic Mean, or WPGMA) which is represented in the heat maps shown in Figures 1A and 1B. As can immediately be seen, with the thresholds used, the number of differentially expressed genes in response to SK&P was 3-fold higher compared with that induced by the control regimen.. Yoga intervention gave rise to 111 differentially expressed genes, whereas this number was 38 for the control regimen, and 14 genes were commonly affected by both yoga and control (Figure 1C). Approximately similar number of genes were up- and down-regulated for the yoga regimen (54 up- and 57 down-regulated genes), whereas in the control regimen there were more down-regulated genes (15 up- and 23 down-regulated genes) (Table S1). The 20 top ranked up- or down-regulated genes are presented in Figure 2. Consistent with the small overlap seen in Figure 1C, there were only two genes (KLF9 and NELL2) which were similarly regulated by yoga and control regimens in the top 20 (Figure 2). The list of all the genes regulated by the two regimens and their overlap are presented in Figure S1.

**Figure 3:**
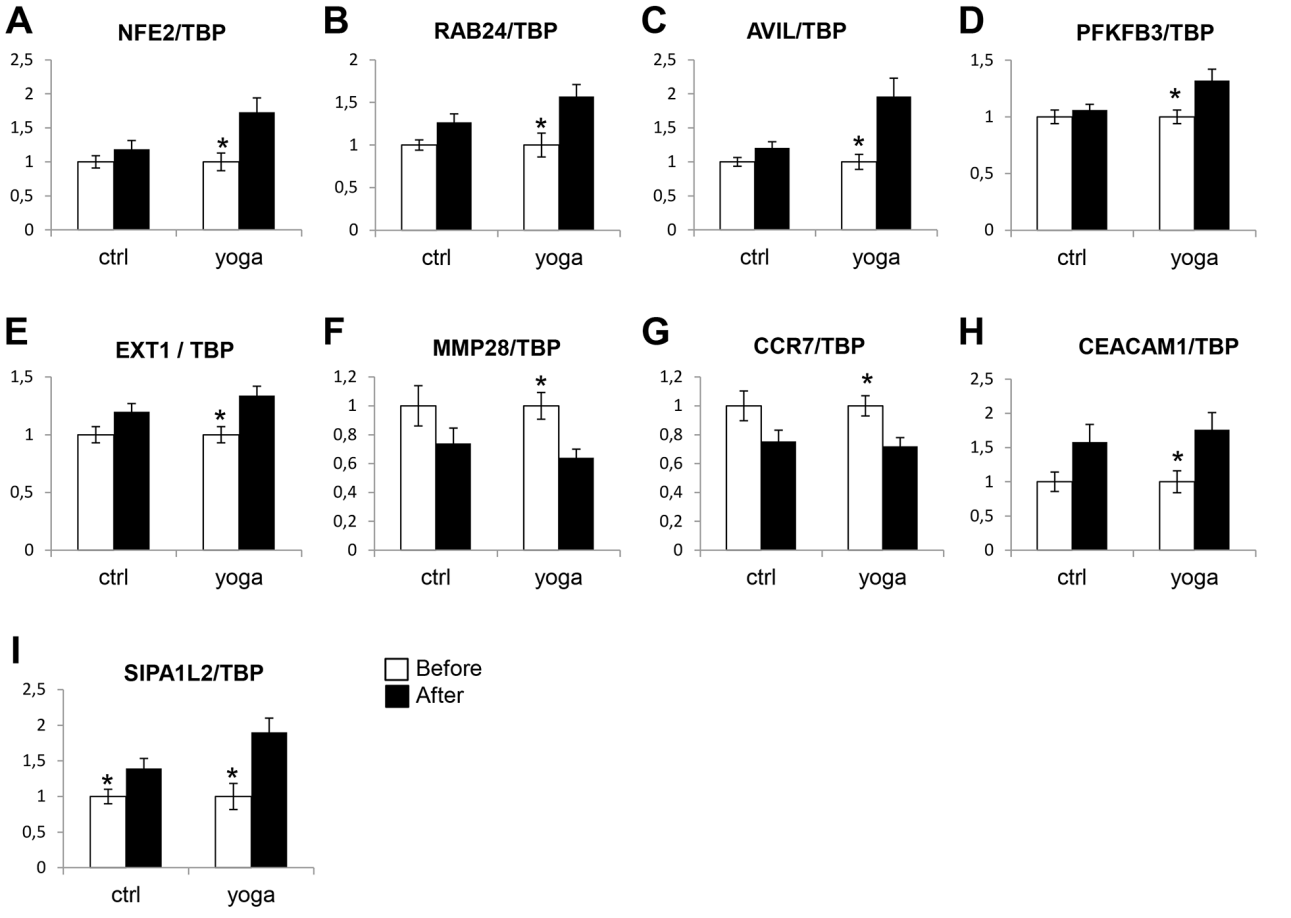
The mRNA expression of the indicated genes which are differentially expressed by the yoga (SK&P), but not the control regimen, according to the microarray data were subjected to qPCR analysis as described in Materials and Methods. Ctrl, control; yoga, SK&P. White and black bars represent samples collected before and after the interventions, respectively. Y axis denotes fold-change in expression. Results represent data from 8 different subjects for each group. Comparisons were made with the student’s T-test. *, *P*<0.05.

**Figure 4:**
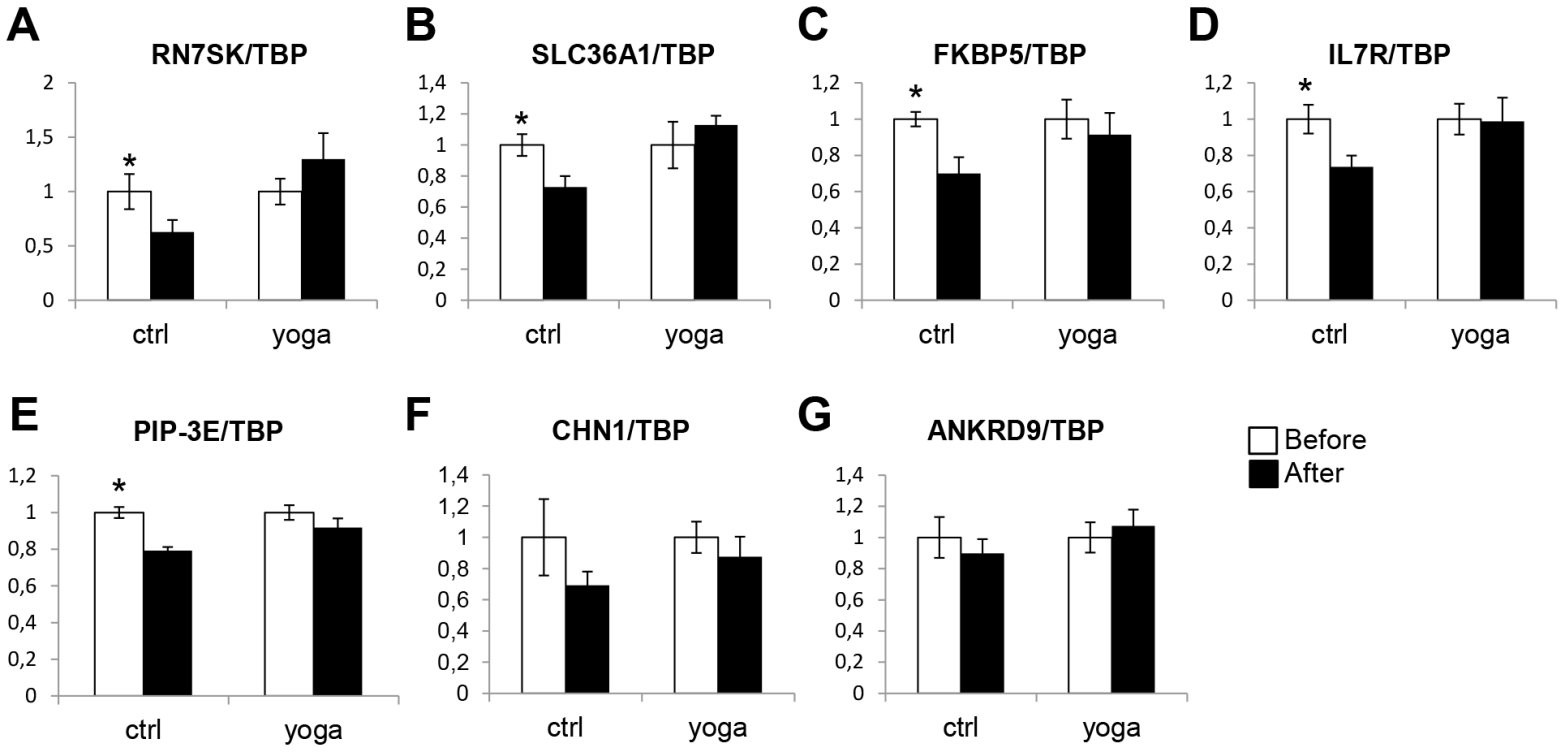
Same as in Figure 3, but a sample of the genes that are differentially expressed by the control, but not the yoga regimen in the microarray analysis, were subjected to qPCR in both the control and SK&P samples. White and black bars represent samples collected before and after the interventions, respectively.

**Figure 5:**
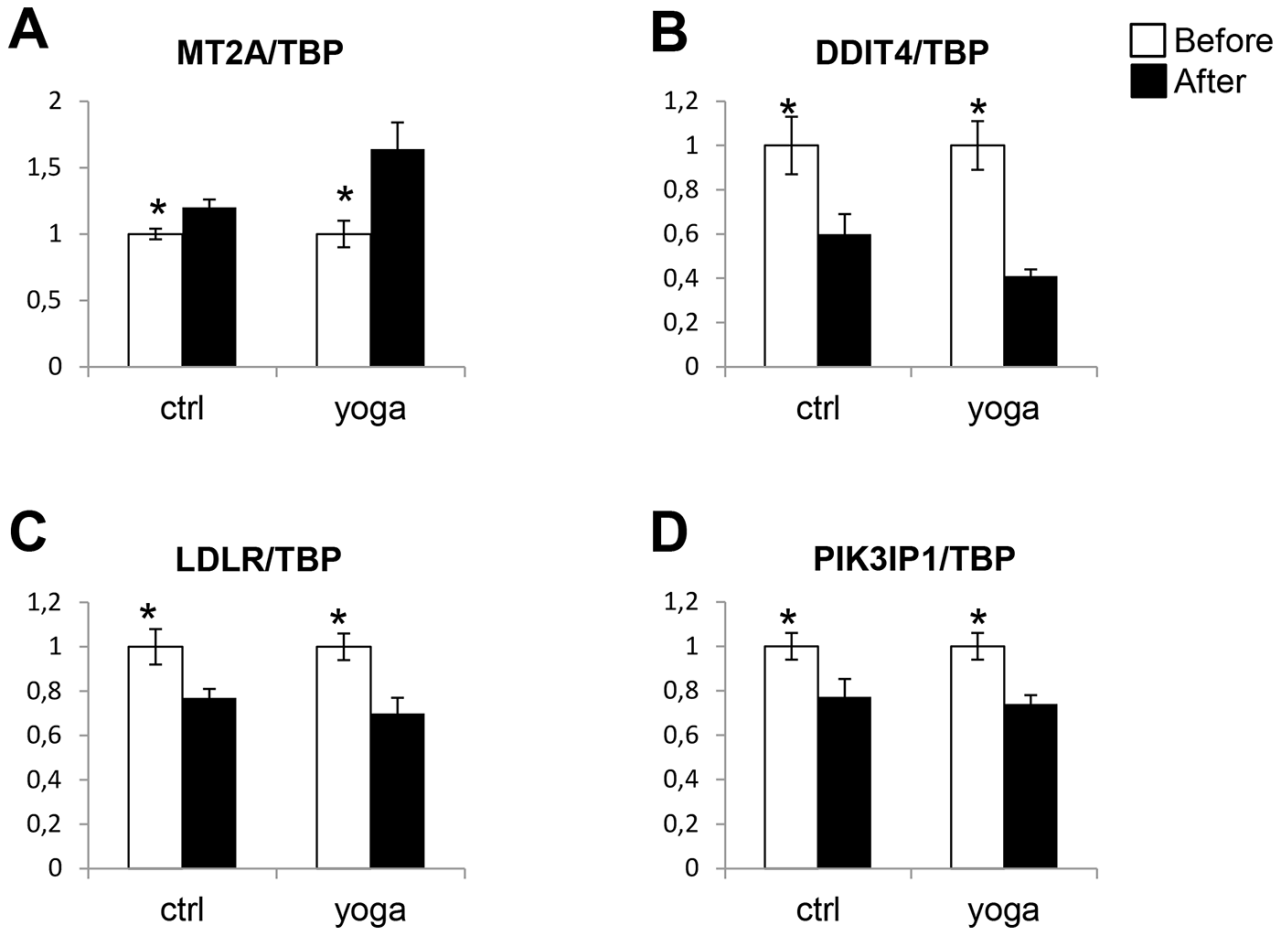
Same as in Figure 3, but a sample of the genes that are differentially expressed by both the control and the yoga regimens according to the microarray analysis were subjected to qPCR. White and black bars represent samples collected before and after the interventions, respectively.

We then sought to validate the gene expression changes indicated by the microarray analysis using quantitative reverse transcription (RT) PCR (qPCR). We first designed primers for 12 genes that were selectively regulated upon SK&P and expression of 9 were detected. As shown in Figure 3A-H, CCR7, AVIL, PFKFB3, CEACAM1, MMP28, NFE2, RAB24, and EXT1 were all differentially regulated by SK&P, but not by the control regimen. The only exception was SIPA1L2 which was upregulated in both groups, but the upregulation was greater in the SK&P group (Figure 3I). Using qPCR, we also checked the validity of differential expression in 7 of the genes in the samples from the control regimen. As shown in Figure 4, 5 of these, RN7SK, SLC36A1, FKBP5, IL7R, PIP-3E, were differentially expressed whereas CHN1 and ANKRD9, although showing trends for change compared with the SK&P group, did not reach significance. We also checked differences in expression of DDIT4, MT2A, LDLR, PIK3IP1 that are similarly affected by both regimens according to the microarray analysis and confirmed this by qPCR (Figure 5). Together, these qPCR data suggest that the results of the microarray analysis are robust and confirm the significant differences on rapid gene expression changes in PBMCs upon practice of SK&P compared with the control regimen.

## Discussion

Here we have shown, to our knowledge for the first time, that there are rapid (within 2 hours of start of practice) and significant gene expression changes in PBMCs of practitioners during a comprehensive yoga program. These data suggest that previously reported effects of yoga practices have an integral physiological component at the molecular level which is initiated immediately during practice and may form the basis for the long term stable effects.

The fact that there were a larger number of genes (approximately 3-fold) which were affected by SK&P compared with the control regimen was consistent with our hypothesis that yoga has specific effects on gene expression in PBMCs. Surprisingly, whereas 97 unique genes were affected by SK&P, only 24 unique genes were affected by the control regimen (Figure 1C). In addition, more than 36% of the genes affected by the control regimen were also influenced by the yoga regimen, indicating that these two regimens to some degree affect similar biological processes. A recent review of comparison studies between yoga and exercise found that yoga may be as effective as, or better than, exercise at improving a variety of health-related outcome measures (for a review, see [24]). Our data are consistent with these earlier findings and suggest that a yoga program may have additional effects over exercise plus simple relaxation in inducing health benefits through differential effects at the molecular level.

Despite the significant changes in gene expression that are induced by the SK&P program, gene ontology analysis by different approaches did not result in the enrichment of specific molecular pathways (data not shown). This suggests that early effects of SK&P on circulating immune cells are quite global and do not engage only specific pathways. Alternatively, the mixture of cells in the PBMC population and the differences in the gene expression patterns induced therein may mask some of the patterns which would otherwise be discovered. It is of interest to note that long term effects of yogic/meditative practices on basal level of gene expression in circulating immune cells did result in the enrichment for certain gene ontology classes in three previous studies [14] [15] [17]. The essential non-overlap between these and the expression patterns that we observe suggests that either a) the specific practices have distinct effects on gene expression, or b) the short term (immediate) effects of yogic practices on gene expression are different than those that are established in the longer term (months or years). It could also be a combination of these factors. Further work incorporating detailed time course analysis (see below) of different practices is required to assess these possibilities.

Examination of the differentially expressed individual genes upon SK&P practice makes it possible to speculate on the cellular effects of the yoga-induced program. For example, the *AVIL*/*ADVILLIN* gene belongs to the gelsolin/villin family of actin regulatory proteins and is highly expressed in the small intestine and the colonic lining with weaker expression in thymus, prostate, testis, and uterus, whereas there is no expression in brain and lung [25]. Our data show that *AVIL* is also expressed in the PBMCs and its expression is significantly increased upon yoga practice. Previous work has found that AVIL regulates ciliogenesis through cytoskeletal actin organization by severing actin filaments [26]. It is tempting to speculate that AVIL may have similar functional roles in PBMCs. For example, accumulation of filamentous actin (F-actin) at the immunological synapse (IS) has been shown to be a prerequisite for the cytotoxic function of natural killer (NK) cells (for a review, see [27]). Reorganization of the actin network is involved in the lytic granule polarization and secretion toward a target cell, a critical aspect of cellular host defense. It is thus possible that yoga practice can activate the directed secretion of lytic granule contents at the IS, and thereby increase NK cell cytotoxicity. This is an appealing hypothesis since previous work has shown that chronic stress can dramatically reduce NK cell cytotoxicity (e.g. [28]) whereas yoga and related practices have been found to be effective antidotes to stress (e.g. [6] [9]). Future molecular and cell biological studies are required to test the validity of this hypothesis.

Another gene that was upregulated by yoga, but not by the control regimen, was *Nuclear Factor Erythroid 2 (NFE2)* which encodes a basic leucine zipper transcription factor that has an essential role in megakaryocyte maturation and platelet production [29]. NFE2 is a master regulator of a number of genes during megakaryocyte differentiation, especially in the later stages, such as proplatelet formation and secretion (for a review, see [30]). Consistently, NFE2 deficient mice lack circulating platelets and die of hemorrhage [29]. Modulators of thrombopoiesis are of considerable interest in hematology since there exist a variety of human thrombocytopenia syndromes. It is therefore tempting to speculate that the increased expression of NFE2 induced by the yoga program may have favorable effects on megakaryocyte maturation and platelet production.

As indicated by the two examples above, it is possible to hypothesize various functional links to the gene products differentially regulated by the yoga and control regimens. However, further work is needed on several fronts. First, since the PBMCs present a mixture of cells which are expected to be differentially affected by the interventions, it is important in future experiments to interrogate subsets of the PBMCs. For example, one could isolate TH1 or TH2 cell fractions, or the NK cell fraction, and do a similar analysis on these cell types. This approach is expected to give results that are specific to the cell type and may uncover changes which are lost due to sampling and changes of multiple cell types. In addition, since these cells have known functions, it will be easier to interpret the data. Furthermore, cell fractionation would control for possible changes in redistribution of leukocyte subsets which has been described to occur during exercise or by mental stress (e.g. [31] [32]). Second, the order of testing was confounded with the intervention content, i.e. in future studies the order of testing should be randomized. Third, it is important to extend the expression changes that are observed at the mRNA level (unless the final gene product is not a protein, but RNA) to the protein level, e.g. through western analysis or ELISA, or better yet, through global proteomics analysis. Fourth, it is essential to carry out cell biological experiments to assess whether the changes in expression lead to functional consequences in the cell of interest. For example, as noted above, AVIL expression which is increased by yoga but not the control regimen, may be involved in regulating NK cell toxicity. This can be directly tested by isolating NK cells before and after the two regimens and checking their activity *in vitro*. These studies can then contribute towards establishing the possible effects of yoga at the molecular and cell biological levels.

It is also desirable to determine how long these effects last and thus longitudinal studies are warranted. These could be of two types: first, blood could be collected in short intervals after the end of the program to see when the observed effects would wane; this can give mechanistic insight to the changes that are observed. Second, one could measure steady state gene expression levels in yoga practitioners compared with controls in a time course of longer periods (months or years). These experiments can then define the transient and stable changes in gene expression over time which can give insights to the mechanisms of action for these practices, from molecular to systemic levels. In these experiments, it is also desirable to increase the sample size and/or study independent set of individuals.

In summary, the data we present show that yogic practices have rapid effects at the molecular level in circulating immune cells. This approach can now be used to more systematically interrogate these molecular changes, define the signals that are triggered by yogic exercises that eventually impact PBMCs, and provide a platform to conduct comparative studies between different yogic practices.

## Supporting Information

Figure S1 **Schematic description of the sample designation for each subject (1 and 3  =  before yoga; 2 and 4  =  after yoga; 5 and 7  =  before the control regimen; 6 and 8  =  after the control regimen).** The regimens are color coded consistent with in Figure 1. The yoga (SK&P) regimen was administered on days 1 and 2, and the control regimen on days 3 and 4, as indicated, on four consecutive days, at the same time of the day and at the same place. The analysis presented is between conditions.
(TIF)

Figure S2 **Principal Component Analysis (PCA) plot.** Each subject has been color-coded and placement of all 8 measurements are indicated, where they tend to cluster together.
(TIF)

Figure S3 **Sequences of the primers used in qPCR experiments are presented.**
(TIF)

Table S1 **Full list of top ranked genes differentially regulated by the yoga or the control regimen presented as in** **Figure 2** **.** The genes that are regulated by both regimens are highlighted in grey.
(TIF)

## References

[1] Barnes PM, Bloom B, Nahin RL (2008) Complementary and alternative medicine use among adults and children: United States, 2007. Natl Health Stat Report: 1–23. 19361005

[2] Nahin RL, Barnes PM, Stussman BJ, Bloom B (2009) Costs of complementary and alternative medicine (CAM) and frequency of visits to CAM practitioners: United States, 2007. Natl Health Stat Report: 1–14. 19771719

[3] Snyderman R, Weil AT (2002) Integrative medicine: bringing medicine back to its roots. Arch Intern Med 162: 395–397. 11863470 10.1001/archinte.162.4.395

[4] Shankar SSR (2010) Patanjali Yoga Sutras: Art of Living Press.

[5] Kuntsevich V, Bushell WC, Theise ND (2010) Mechanisms of yogic practices in health, aging, and disease. Mt Sinai J Med 77: 559–569. 20960557 10.1002/msj.20214

[6] Pilkington K, Kirkwood G, Rampes H, Richardson J (2005) Yoga for depression: the research evidence. J Affect Disord 89: 13–24. 16185770 10.1016/j.jad.2005.08.013

[7] Pullen PR, Nagamia SH, Mehta PK, Thompson WR, Benardot D, et al (2008) Effects of yoga on inflammation and exercise capacity in patients with chronic heart failure. J Card Fail 14: 407–413. 18514933 10.1016/j.cardfail.2007.12.007

[8] Janakiramaiah N, Gangadhar BN, Naga Venkatesha Murthy PJ, Harish MG, Subbakrishna DK, et al (2000) Antidepressant efficacy of Sudarshan Kriya Yoga (SKY) in melancholia: a randomized comparison with electroconvulsive therapy (ECT) and imipramine. J Affect Disord 57: 255–259. 10708840 10.1016/s0165-0327(99)00079-8

[9] Kjellgren A, Bood SA, Axelsson K, Norlander T, Saatcioglu F (2007) Wellness through a comprehensive yogic breathing program - a controlled pilot trial. BMC Complement Altern Med 7: 43. 18093307 10.1186/1472-6882-7-43 PMC2231388

[10] Sharma H, Sen S, Singh A, Bhardwaj NK, Kochupillai V, et al (2003) Sudarshan Kriya practitioners exhibit better antioxidant status and lower blood lactate levels. Biol Psychol 63: 281–291. 12853172 10.1016/s0301-0511(03)00071-1

[11] Bhatia M, Kumar A, Kumar N, Pandey RM, Kochupillai V (2003) Electrophysiologic evaluation of Sudarshan Kriya: an EEG, BAER, P300 study. Indian J Physiol Pharmacol 47: 157–163. 15255618

[12] Garland EL, Howard MO (2009) Neuroplasticity, psychosocial genomics, and the biopsychosocial paradigm in the 21st century. Health Soc Work 34: 191–199. 19728478 10.1093/hsw/34.3.191 PMC2933650

[13] Cole SW (2010) Elevating the perspective on human stress genomics. Psychoneuroendocrinology 35: 955–962. 20630660 10.1016/j.psyneuen.2010.06.008 PMC2917592

[14] Li QZ, Li P, Garcia GE, Johnson RJ, Feng L (2005) Genomic profiling of neutrophil transcripts in Asian Qigong practitioners: a pilot study in gene regulation by mind-body interaction. J Altern Complement Med 11: 29–39. 15750361 10.1089/acm.2005.11.29

[15] Dusek JA, Otu HH, Wohlhueter AL, Bhasin M, Zerbini LF, et al (2008) Genomic counter-stress changes induced by the relaxation response. PLoS One 3: e2576. 18596974 10.1371/journal.pone.0002576 PMC2432467

[16] Sharma H, Datta P, Singh A, Sen S, Bhardwaj NK, et al (2008) Gene expression profiling in practitioners of Sudarshan Kriya. J Psychosom Res 64: 213–218. 18222135 10.1016/j.jpsychores.2007.07.003

[17] Black DS, Cole SW, Irwin MR, Breen E, St Cyr NM, et al.. (2012) Yogic meditation reverses NF-kappaB and IRF-related transcriptome dynamics in leukocytes of family dementia caregivers in a randomized controlled trial. Psychoneuroendocrinology. 10.1016/j.psyneuen.2012.06.011 PMC3494746 22795617

[18] Dysvik B, Jonassen I (2001) J-Express: exploring gene expression data using Java. Bioinformatics 17: 369–370. 11301307 10.1093/bioinformatics/17.4.369

[19] Yeung KY, Ruzzo WL (2001) Principal component analysis for clustering gene expression data. Bioinformatics 17: 763–774. 11590094 10.1093/bioinformatics/17.9.763

[20] Tusher VG, Tibshirani R, Chu G (2001) Significance analysis of microarrays applied to the ionizing radiation response. Proc Natl Acad Sci U S A 98: 5116–5121. 11309499 10.1073/pnas.091062498 PMC33173

[21] Breitling R, Armengaud P, Amtmann A, Herzyk P (2004) Rank products: a simple, yet powerful, new method to detect differentially regulated genes in replicated microarray experiments. FEBS Lett 573: 83–92. 15327980 10.1016/j.febslet.2004.07.055

[22] Klokk TI, Kilander A, Xi Z, Waehre H, Risberg B, et al (2007) Kallikrein 4 is a proliferative factor that is overexpressed in prostate cancer. Cancer Res 67: 5221–5230. 17545602 10.1158/0008-5472.CAN-06-4728

[23] Astin JA, Shapiro SL, Eisenberg DM, Forys KL (2003) Mind-body medicine: state of the science, implications for practice. J Am Board Fam Pract 16: 131–147. 12665179 10.3122/jabfm.16.2.131

[24] Ross A, Thomas S (2010) The health benefits of yoga and exercise: a review of comparison studies. J Altern Complement Med 16: 3–12. 20105062 10.1089/acm.2009.0044

[25] Marks PW, Arai M, Bandura JL, Kwiatkowski DJ (1998) Advillin (p92): a new member of the gelsolin/villin family of actin regulatory proteins. J Cell Sci 111 (Pt 15): 2129–2136. 10.1242/jcs.111.15.2129 9664034

[26] Kim J, Lee JE, Heynen-Genel S, Suyama E, Ono K, et al (2010) Functional genomic screen for modulators of ciliogenesis and cilium length. Nature 464: 1048–1051. 20393563 10.1038/nature08895 PMC2929961

[27] Orange JS (2008) Formation and function of the lytic NK-cell immunological synapse. Nat Rev Immunol 8: 713–725. 19172692 10.1038/nri2381 PMC2772177

[28] Glaser R, Rice J, Speicher CE, Stout JC, Kiecolt-Glaser JK (1986) Stress depresses interferon production by leukocytes concomitant with a decrease in natural killer cell activity. Behav Neurosci 100: 675–678. 2430594 10.1037//0735-7044.100.5.675

[29] Shivdasani RA, Orkin SH (1995) Erythropoiesis and globin gene expression in mice lacking the transcription factor NF-E2. Proc Natl Acad Sci U S A 92: 8690–8694. 7567998 10.1073/pnas.92.19.8690 PMC41032

[30] Shivdasani RA (2001) Molecular and transcriptional regulation of megakaryocyte differentiation. Stem Cells 19: 397–407. 11553848 10.1634/stemcells.19-5-397

[31] Shinkai S, Shore S, Shek PN, Shephard RJ (1992) Acute Exercise and Immune Function - Relationship between Lymphocyte Activity and Changes in Subset Counts. International Journal of Sports Medicine 13: 452–461. 1428375 10.1055/s-2007-1021297

[32] Nieman DC, Simandle S, Henson DA, Warren BJ, Suttles J, et al (1995) Lymphocyte Proliferative Response to 2.5 Hours of Running. International Journal of Sports Medicine 16: 404–409. 7591393 10.1055/s-2007-973028

