# Supplementary figures and images for: Rapid Gene Expression Changes in Peripheral Blood Lymphocytes upon Practice of a Comprehensive Yoga Program
Source: PLoS One. 2013 Apr 17;8(4):e61910. doi: 10.1371/journal.pone.0061910 (PMC3629142; doi:10.1371/journal.pone.0061910)

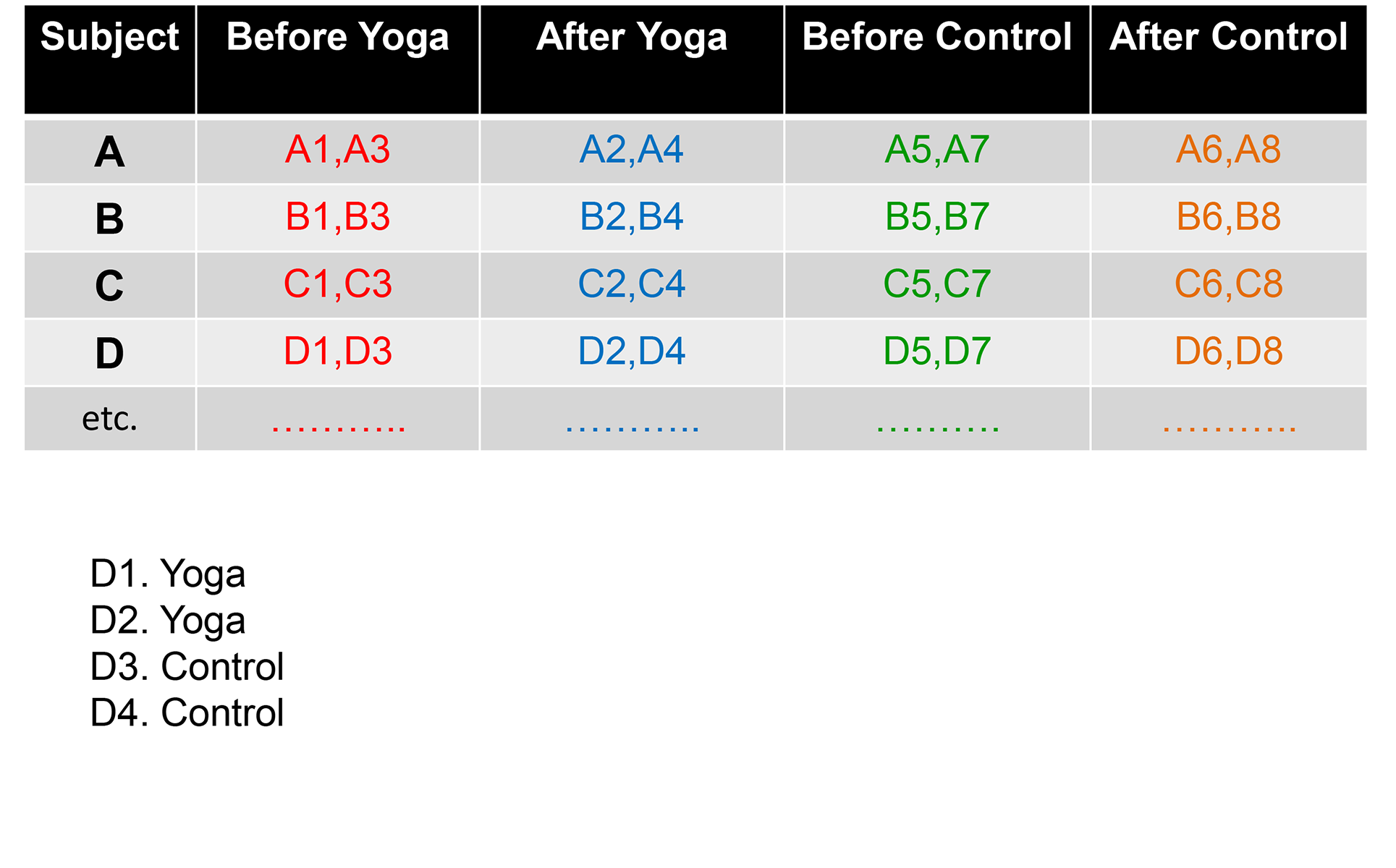

Supplement: Figure S1 — Schematic description of the sample designation for each subject (1 and 3 = before yoga; 2 and 4 = after yoga; 5 and 7 = before the control regimen; 6 and 8 = after the control regimen). The regimens are color coded consistent with in Figure 1. The yoga (SK&P) regimen was administered on days 1 and 2, and the control regimen on days 3 and 4, as indicated, on four consecutive days, at the same time of the day and at the same place. The analysis presented is between conditions. (TIF) [file pone.0061910.s001.tif]

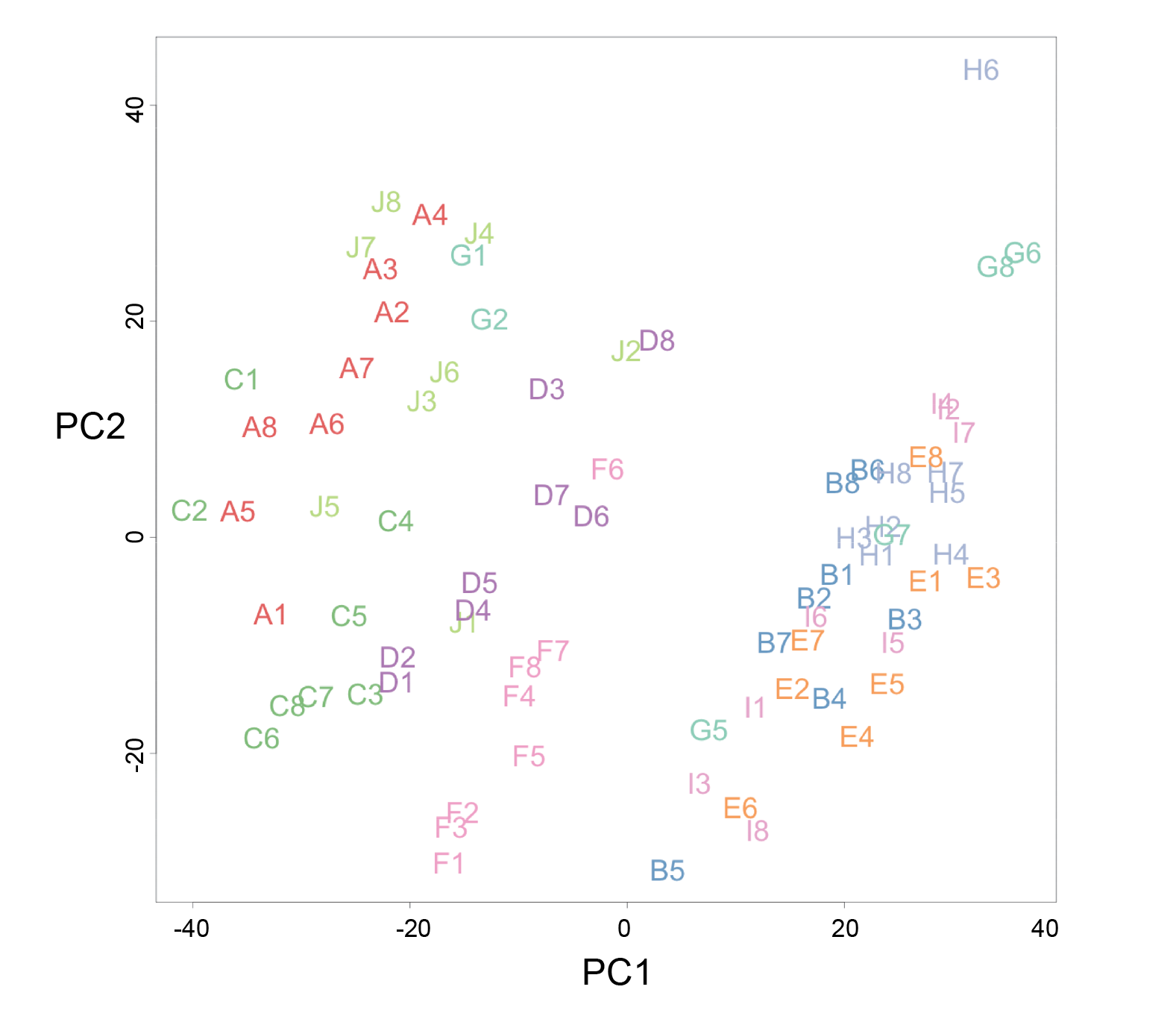

Supplement: Figure S2 — Principal Component Analysis (PCA) plot. Each subject has been color-coded and placement of all 8 measurements are indicated, where they tend to cluster together. (TIF) [file pone.0061910.s002.tif]

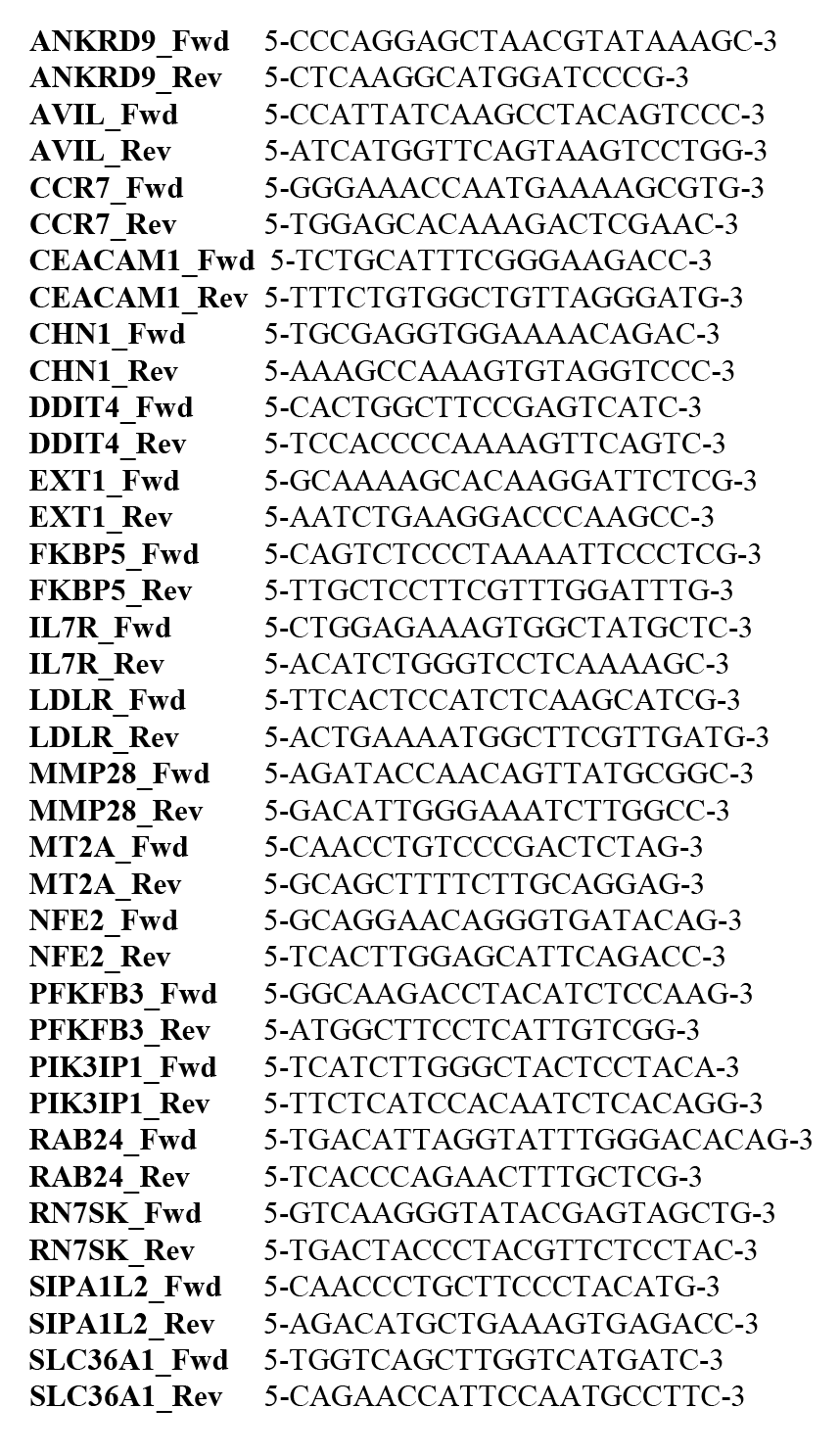

Supplement: Figure S3 — Sequences of the primers used in qPCR experiments are presented. (TIF) [file pone.0061910.s003.tif]

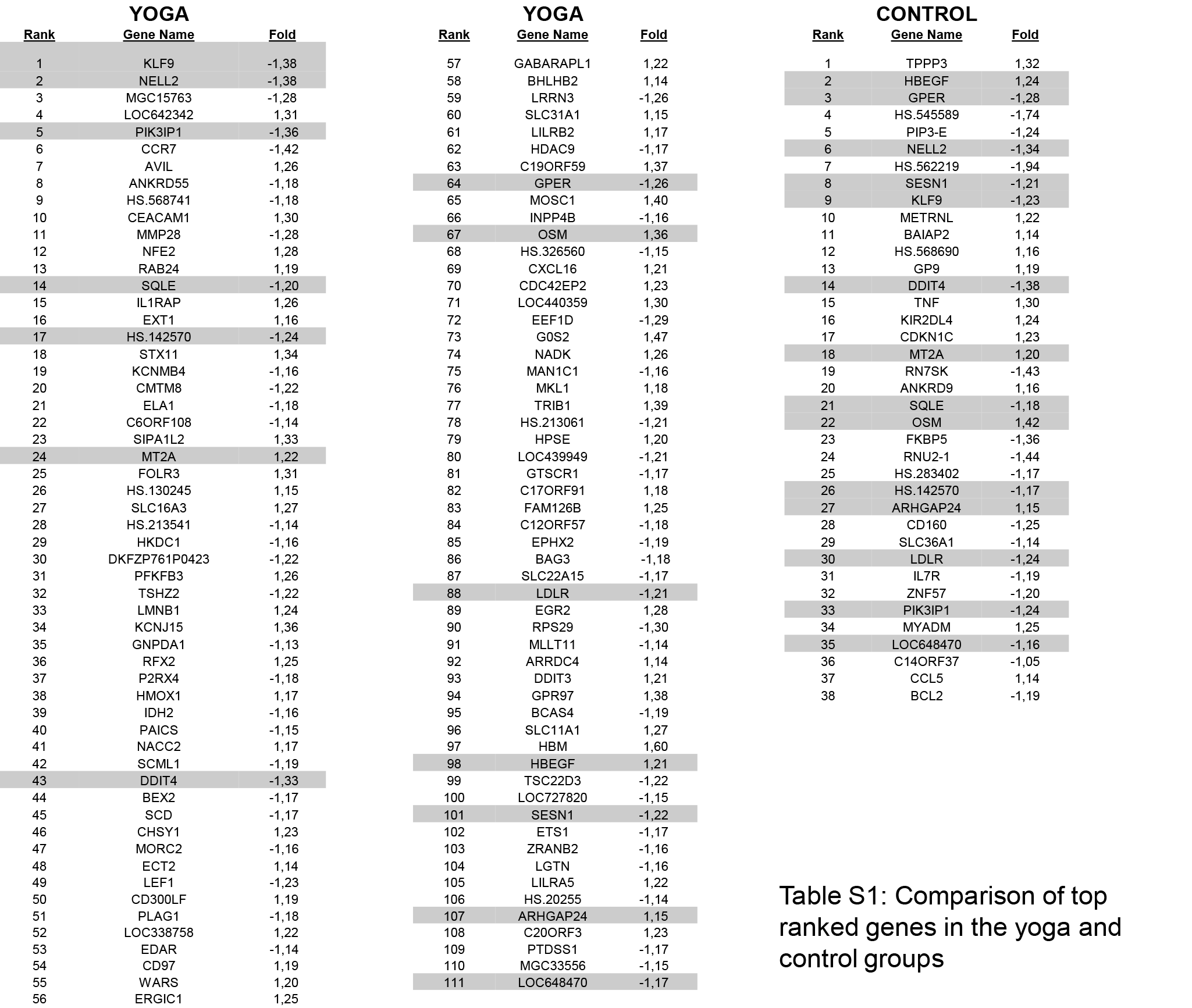

Supplement: Table S1 — Full list of top ranked genes differentially regulated by the yoga or the control regimen presented as in Figure 2 . The genes that are regulated by both regimens are highlighted in grey. (TIF) [file pone.0061910.s004.tif]
